# Optimization of intestinal microsomal preparation in the rat: A systematic approach to assess the influence of various methodologies on metabolic activity and scaling factors

**DOI:** 10.1002/bdd.2070

**Published:** 2017-04-18

**Authors:** Oliver J.D. Hatley, Christopher R. Jones, Aleksandra Galetin, Amin Rostami‐Hodjegan

**Affiliations:** ^1^ CertaraBlades Enterprise CentreSheffieldS2 4SUUK; ^2^ DMPKHeptares TherapeuticsWelwyn Garden CityAL7 3AXUK; ^3^ Centre for Applied Pharmacokinetic ResearchUniversity of ManchesterManchesterM13 9PTUK

**Keywords:** intestinal metabolism, scaling factors, *in vitro–in vivo* extrapolation

## Abstract

The metabolic capacity of the intestine and its importance as the initial barrier to systemic exposure can lead to underestimation of first‐pass, and thus overestimation of oral bioavailability. However, the *in vitro* tools informing estimates of *in vivo* intestinal metabolism are limited by the complexity of the *in vitro* matrix preparation and uncertainty with the scaling factors for *in vitro* to *in vivo* extrapolation. A number of methods currently exist in the literature for the preparation of intestinal microsomes; however, the impact of key steps in the preparation procedure has not been critically assessed. In the current study, changes in enterocyte isolation, the impact of buffer constituents heparin and glycerol, as well as sonication as a direct method of homogenization were assessed systematically. Furthermore, fresh vs. frozen tissue samples and the impact of microsome freeze thawing was assessed. The rat intestinal microsomes were characterized for CYP content as well as metabolic activity using testosterone and 4‐nitropheonol as probes for CYP and UGT activity, respectively. Comparisons in metabolic activity and scaled unbound intestinal intrinsic clearance (*CL*_intu,gut_) were made to commercially available microsomes using 25 drugs with a diverse range of metabolic pathways and intestinal metabolic stabilities. An optimal, robust and reproducible microsomal preparation method for investigation of intestinal metabolism is proposed. The importance of characterization of the *in vitro* matrix and the potential impact of intestinal scaling factors on the *in vitro–in vivo* extrapolation of *F*_G_ needs to be investigated further. © 2017 The Authors *Biopharmaceutics & Drug Disposition* Published by John Wiley & Sons Ltd.

## Introduction

The bioavailability (*F*_oral_) of each orally administered drug is uniquely determined by the varying extent of release from the formulation, dissolution and permeation through the gut wall, and the respective fractions escaping first‐pass metabolism within the intestine (*F*_G_) and the liver (*F*_H_) 1, 2. Given the metabolic capacity of the intestine and its importance as the initial barrier to systemic exposure, ignoring its role in first‐pass drug elimination can lead to underestimation of first‐pass and thus overestimation of *F*_oral_ 3, 4. Furthermore, failure to incorporate a mechanistic understanding of this process may lead to unexpected changes in exposure through: intestinal‐specific interactions, e.g. CYP3A inhibition by grapefruit juice 5; disease populations, e.g. reduced intestinal CYP3A4 in celiac patients 6; or through drastic surgical interventions, e.g. gastric‐bypass 7, 8. Prior to best candidate drug selection, an assessment of the predicted *F*_oral_, based on *in vitro* permeability and metabolism experiments, forms a key step in determining potential issues with exposure as well as the selection of appropriate doses and formulation strategies 2, 3.

The *in vitro* tools informing estimates of *F*_G_ therefore need to be *in vivo* representative, reproducible and reliable to facilitate extrapolation of data to the *in vivo* situation. However, in the case of intestinal metabolism, the complexity of the *in vitro* matrix preparation, uncertainty with the *in vitro* to *in vivo* scaling factors (in particular for non‐P450 substrates), and limitations in quantifying the absolute contribution *in vivo* can result in poor confidence in these estimates 3, 9, 10, 11, 12. This may play a significant role in the ability to predict exposures in early discovery 13 and bring insight to species differences in metabolism, which can result in discrepancies between animal and human bioavailability 14. Understanding these at the early stages helps with the plan of studies for later development.

Intestinal microsomes constitute a convenient *in vitro* matrix for the assessment of intestinal metabolism 3, 9, 10, 11, 15. However, the different isolation techniques employed contribute to a variable quality of the *in vitro* preparation 9. The purpose of this work therefore was to assess critically and systematically the key steps in the preparation of intestinal microsomes, using the rat as a model species. The impact of changes to the homogenization steps and buffer constituents were investigated in order to arrive at an optimized methodology for intestinal microsome preparation and isolation.

## Materials and Methods

### Reagents

All laboratory chemicals were purchased from Sigma (Dorset, UK) unless detailed in the text.

### Animals

Rat intestinal microsomes were prepared in‐house at AstraZeneca, Alderley Park, UK. The protocol and procedures employed conformed to UK legislation under the Animals (Scientific Procedures) Act 1986 Amendment Regulations (SI 2012/3039). Since intestinal microsome yields are low, intestinal microsomes were pooled each day from three rats to provide higher yields, without compromising the preparation time and therefore the microsomal quality. Male albino Han‐Wistar (Harlan, UK), 289 ± 21 g, ranging from 9 to 10 weeks were euthanized by rising CO_2_ at approximately the same time each day (8.30–9.30 a.m.). The animals were either redundant to an on‐going project related pharmacokinetic study (due to age) and/or utilized from other on‐going in‐house studies requiring other organs, and as such not solely killed for the removal of the intestine. Animals were not subject to any compound administration for at least 1 week prior to use. Although unlikely, it was not possible to rule out any effects from prior studies that may affect intestinal metabolism. Animals were bled prior to organ procurement in order to remove proteases present in the blood, thereby reducing the risk of damage to intestinal CYPs, since the intestine (like the liver) is highly perfused 16. Death was confirmed by cervical dislocation, and the first 60 cm of intestine proximal to the pylorus was removed. The intestinal length of 60 cm (approximately 50% of the total rat intestinal length 17 was selected as the most routinely utilized length in the literature to allow for method comparison 16, 18, 19, 20. Since the highest CYP content reported has been in the proximal end of the intestine (duodenum and jejunum) 21, there would be an improved chance of CYP quantification.

### Intestinal tissue preparation

Following extraction, the intestine was flushed to remove food material and excess mucus, using a wash buffer solution (pH 7.4) consisting of 0.9% *w*/*v* NaCl (Fischer Scientific, UK) and 0.5 mm dithiothreitol (DTT) – which was used to prevent degradation of CYP. Furthermore, a protease inhibitor (PI) cocktail (0.1% *v*/v, used at the same concentration in all subsequent solutions) was added to prevent protease damage to CYP. Whilst this cocktail is known to have inhibitory potential to rat cyp2d (incorrectly reported as cyp2b in publication) 16, it is reported that cyp2d plays a more significant role in the lower parts of the intestine 22. All solutions were prepared the day prior to isolation (with the exception of DTT, which was added immediately before use) and stored at 4°C. Excess fat on the outside of the intestine was then removed and the remaining intestinal samples weighed.

### Optimization of intestinal microsomal preparation

On transfer to the laboratory, the intestinal lumen was flushed using a syringe with 30 ml of solution A, pH 7.4, consisting of phosphate buffered solution (PBS) buffer (used in all subsequent preparation buffers), 27 mm sodium citrate monobasic, 0.5 mm DTT and PI. Sodium citrate, a mild chelating agent, was used to promote cell dissociation prior to elution 23. The intestinal lumen was then filled with solution A and re‐clamped, and incubated on ice in a trough of solution A for 30 min. The entire procedure was performed at 4°C to limit warm ischaemia and proteolysis.

**Figure 1:**
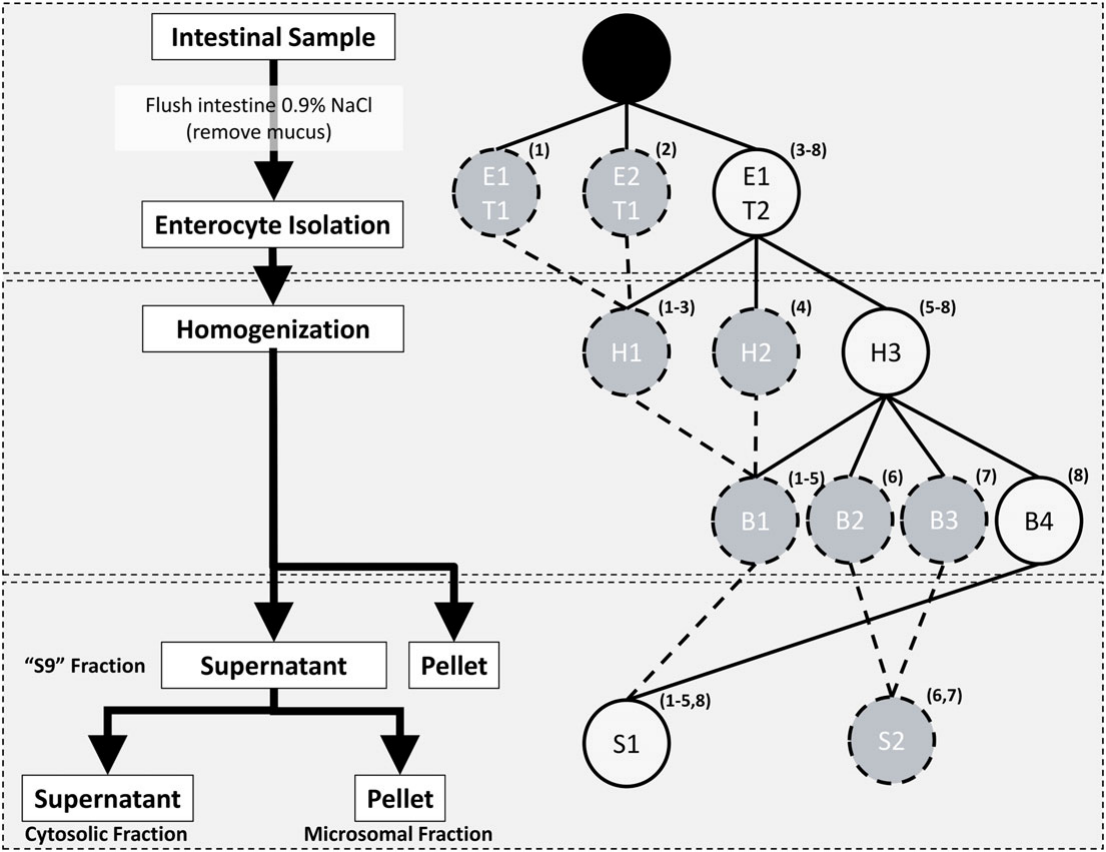
Summary of method and optimization steps for optimizing preparations of rat intestinal microsomes. E1, ETDA 5 mm; E2, EDTA 1.5 mm; T1. 60 min elution; T2, 20 min elution; H1, 6 W sonication; H2, 18 W sonication; H3, 30 W sonication; B1, no heparin or glycerol; B2, glycerol 20% *v*/v; B3, heparin 3 U/ml, glycerol 20% *v*/v; B4, heparin 9 U/ml. S1, no glycerol; S2, glycerol 20% *v*/v. Solid lines represent progression of optimized method, dashed lines represent sub‐optimal method combinations. The applicable preparation method number in relation to Table 1 is shown within each circle. Each preparation method represents three independent preparations of three pooled intestinal samples

The method for intestinal microsome preparation via elution was based on the method of Fasco *et al*. 20, however, three additional key steps based on differing reports in the literature were chosen for optimization: elution procedure; homogenization protocol; and the effect of addition of two buffer constituents: glycerol and/or heparin. Therefore, a total of eight preparation methods were assessed. A summary of the conditions tested for intestinal microsome preparation is shown in Figure 1.

### Enterocyte elution conditions

Enterocytes were extracted by EDTA based calcium elution. EDTA binding to calcium disrupts the joining of cadherins responsible for cell adhesion, and allows cell detachment 24. Solution B (pH 7.4) contained PBS, EDTA, 0.5 mm DTT and PI with 3 UI/ml heparin. Heparin was used to reduce protein trapping by mucus and therefore to improve pellet formation. For incubation varying EDTA concentrations of 1.5 mm vs*.* 5 mm were used.

Following incubation in solution A, the intestines were flushed with solution B to flush solution A for collection. Upon filling the intestine with approximately 20 ml of solution B, both ends of the intestine were clamped shut, and the intestine was placed in a conical flask containing an incubation buffer solution (pH 7.2) consisting of PBS and 20% *v*/v glycerol and then agitated on ice at 250 rpm (Orbital Shaker SSL1, Stuart) for an initial 20 min. Solution B was drained into a conical flask and the intestines flushed with another 10 ml of solution B, clamped and re‐agitated for a further 20 min. This process was repeated two more times. In other experiments that used a shorter total incubation time of 20 min, the intestine was flushed with solution B following a 10 min and two 5 min incubations.

### Investigations of homogenization buffer constituents

In order to minimize mucus contamination, the total combined raw pool of intestinal mucosa (containing enterocytes) from the three incubations in solution B was centrifuged at 2000×*g* (Sorvall legend RT centrifuge, Thermo Fisher Scientific) for 10 min in pre‐weighed centrifuge tubes, as described in a previously published account 20. This procedure was repeated twice to wash the pellet in solution C. Following the final centrifugation, the pellet was weighed (mucosal weight) and reconstituted in 3 ml of solution C per g of cells. The homogenization buffer (pH 7.4) contained PBS, 0.25 m sucrose, 0.5 mm EDTA, 5 mm histidine and PI. Disruption of the cells was achieved using a Potter‐Elvejhem rotating homogenizer (10 passes, 1250 rpm) (IKA KS130 Basic). The homogenate was treated with an ultrasonic probe (VCX130PB, vibracell, Sonics, Newtown, USA) for two 10 s burst (30 s between each burst), using a 10–25 ml tip to disrupt the cell membranes. The resultant mixture was re‐homogenized using the Potter‐Elvehjem. A 4 ml sample of homogenate was then collected and utilized for protein and CYP determinations for an assessment of the extent of procedural losses and the necessary correction factor values for extrapolation from *CL*_int_ experiments. The homogenate was spun at 1000×*g* for 4 min three times, washing the pellet in solution C and collection of the supernatant.

The homogenate was ultracentrifuged at 10000×*g* (Optima LE‐80 K, Beckman Coulter, 50.2Ti rotor) for 15 min to pellet the mitochondria, peroxisomes, intact cells, lysosomes and nuclei. The ‘S9’ supernatant was filtered through NYTAL filter mesh (pore size: 150 μm) (Lockertex, Warrington, UK) in order to trap and prevent transfer of a mucosal layer formed on top of the supernatant, and then ultracentrifuged for 70 min at 100000×*g*. The final pellet was re‐homogenized with 10 passes on ice in a 5 ml Potter‐Elvehjem homogenizer in Tris‐hydrochloric acid buffer (pH 7.4) containing PI with or without 20% *v*/v glycerol. Microsomal samples were stored on ice (for immediate protein and CYP content and activity marker analysis) and the remaining yield was stored at −80°C.

### Impact of homogenization on intestinal microsome isolation

When investigating the effect of homogenization conditions on the microsomal yield the amplitude of the sonication was assessed at 20% (6 W), 60% (18 W) and 100% (30 W).

### Assessment of the heparin and glycerol effect

The effect of the addition of glycerol (20% *v*/v) to the homogenization buffer (solution C) and the storage buffer (solution D) was investigated. Glycerol is routinely used with the liver microsome preparation 25 and other extrahepatic microsomes e.g. lung 26, since it is reported to protect CYP during homogenization 27. Furthermore the addition of heparin (3 U/ml) to the homogenization and centrifugation buffer (solution C) was tested to attempt to increase the microsomal yield since it prevents aggregation caused by mucus contamination 27. Finally, the impact of using 9 U/ml heparin in the incubation buffer (solution B) was investigated, using no glycerol or heparin in solution C. All experiments investigating the effects of glycerol and heparin were undertaken following homogenization at 100% amplitude (30 W) sonication and 5 mm EDTA.

### Robustness of method and impact of tissue preparation

Microsomes prepared previously via the optimized method (preparation method 8) were combined to form pool 1 (*n* = 9 rats). A second set of three preparations was combined to form pool 2 (*n* = 9 rats). Intestinal microsomes were also prepared from intestinal tissue flash‐frozen in tubes on arrival in the laboratory and kept at −80°C until thawing on ice on the morning of preparation, (*n* = 3 occasions of *n* = 3 samples). In addition, for comparative purposes microsomes were also prepared from scraped intestinal tissues. Microsomes were prepared after cutting along the length of intestine and gently scraping the intestinal epithelial with a glass microscope slide (*n* = 1 occasion, *n* = 3 samples). Commercially available pooled intestinal microsomes were also used as a comparator to those produced in‐house. Han Wistar (HW) (R6000.I, lot #0810335, 90 male donor pool), and Sprague–Dawley (SD) (R1000.I, lot #1010043, 135 male donor pool) were obtained from Xenotech (Tebu‐Bio Ltd, Peterborough, Cambridgeshire, UK). The microsomes were stored at −80°C prior to experiments.

### Determination of protein recovery and correction for losses

Protein concentrations of both homogenate and microsome samples were quantified against bovine serum albumin (BSA) assessed using the bicinchoninic acid (BCA) assay (Pierce Biotechnology, IL, USA) based on the method of Smith *et al*. 28. Loss of microsomal protein during each preparation method was assessed using both the total protein and the absolute CYP content. Following method development, the robustness of the scaling factors was assessed for further preparations using additional microsomal CYP and UGT activity markers.

### Determination of cytochrome P450 content

A Shimadza UV‐24001 double beam spectrophotometer was used to measure the CYP content in the intestinal homogenate and microsomal samples. Measurement of CYP was achieved using the method of dithionate‐difference spectroscopy 29, where the difference between the CO‐complex of ferrous CYP and the oxidized pigment are determined. The method of the reduced minus oxidized difference spectrum method 30 was not used in order to avoid interference by any residual haemoglobin that might have contaminated the samples as described previously 25. Homogenate and microsomal preparations were measured as per Wilson *et al*. 25, except that this solution was at pH 7.4 rather than pH 7.25. The concentration of CYP in the sample was determined using Equation (1). All samples were analysed in triplicate.

(1) $$\text{nmolCYP}.{\mathrm{mg}}^{-1}=\frac{\Delta \;{\text{Absorbance}}_{450-490\;\mathrm{nm}}\;}{\varepsilon_{450-490}{\mathrm{mM}}^{-1}{\mathrm{cm}}^{-1}.\left[\text{Protein}\;\left(\mathrm{mg}.{\mathrm{ml}}^{-1}\right)\right]}$$

Where ε_450–490_ = 104 mm/cm 29.

### Recovery and correction for losses

The microsomal specific marker based on the measured CYP content was utilized as a measure of loss (recovery factor) of microsomal protein 25, 31, 32. Recovery was calculated in a similar fashion to the referenced studies; excepting that the total protein content in the homogenate was also corrected for the sample of homogenate removed prior to ultracentrifugation, to increase the precision in the recovery estimate (Eq. (2)). Corrected measures of microsomal protein (mg) were normalized for mucosal yield (g wet weight) determined using Equation (3).

(2) $$\text{Recovery Factor}=\frac{{\text{nmoles}\;\mathrm{CYP}}_{\mathrm{mic}}\times {{\mathrm{mg}\;\text{Protein}}_{\mathrm{mic}}}^{-1}\times {\text{total}\;\mathrm{mg}\;\text{Protein}}_{\mathrm{mic}\;}\;}{\text{nmoles}{\mathrm{CYP}}_{\mathrm{hom}}\times {{\mathrm{mg}\;\text{Protein}}_{\mathrm{hom}}}^{-1}\times \left({\text{total}\;\mathrm{mg}\;\text{Protein}}_{\mathrm{hom}}-{\mathrm{mg}\;\text{protein removed}\;}_{\mathrm{hom}}\right)}$$

where hom is homogenate and mic is microsomes.

(3) $$\text{Corrected MPPGM}=\frac{\mathrm{mg}\;{\text{Protein}}_{\mathrm{mic}\;}\times g\;\mathrm{wet}\;\text{weight mucosoal}\;{\text{protein}}^{-1}}{\text{Recovery Factor}}\;$$

### Assessment of CYP activity in rat intestinal microsomes

The CYP enzyme activities were determined for the in‐house and commercial intestinal microsomes using the probe substrate testosterone. Testosterone metabolites and the respective rat cyp isoforms responsible for their formation are shown in Supplemental material Table 1 33. The formation of all testosterone metabolites was monitored following incubations with rat intestinal microsomes at 1 mg/ml 0.1 m phosphate buffer (pH 7.4) and a final test concentration of 100 μm testosterone (internal AZ compound inventory stock) (2‐fold human *K*_m_) 34, 35 as assumed for mouse intestinal microsomes in previous reports 36. Rat intestinal microsomes were incubated in triplicate with 1 mm NADPH (#481973, Calbiochem, San Diego, USA), pre‐warmed for 5 min at 37°C and shaken at 900 rpm using a CAT SH10 Heater shaker (Hamiliton robotics, Reno, Nevada, USA). The reactions were initiated by the addition of testosterone. Samples were taken at 5, 15, 30 and 60 min. Linearity was observed for the major formed metabolites up to 30 min. Following optimization of the bioanalytical methodology to improve reproducibility and sensitivity based on increasing the efficiency of protein precipitation 37, the samples were quenched 1:2 (sample: quench) in ice cold acetonitrile containing internal standard (1 μm 11‐β OH TEST) and stored overnight at −20°C. The following day, the plates were spun at 3000 rpm for 15 min, and 50 μl supernatant was diluted in 200 μl UPH_2_O. Samples were quantified using a Waters Acquity UPLC system with a PDA coupled to a G2 Q‐ToF MS (Waters, Milford, MA, USA), and a Acquity UPLC BEH C18 column, 130 Å, 1.7 μm, 2.1 mm × 100 mm and Acquity UPLC column with an in‐Line Filter Kit made up of the same phase (Waters). The sample (25 μl) was detected using an electrospray interface in positive mode, and source and desolvation temperatures, and desolvation and cone gas flows were 120°C and 400°C, and 800 l/h and 20 l/h, respectively. The cone voltage was 30 V. The elution times and LLOQ of testosterone and its hydroxy metabolites are shown in Supplemental material Table 2. The quantification of testosterone metabolites listed in Supplemental material Table 1 was made using standard curves (5–5000 pmol/ml) prepared from no cofactor added microsomes and prepared identically to the assay samples.

### Assessment of glucuronidation in rat intestinal microsomes

The UGT activity via measure of 4‐nitrophenol glucuronide formation (4‐NP gluc) was determined using in‐house and also commercial rat intestinal microsomes. Metabolite formation was monitored as before following incubations with microsomes (1 mg/ml) in 0.1 m phosphate buffer (pH 7.4) at a final test concentration of 100 μm 4‐NP concentration (>2‐fold human *K*_m_) 35. Microsomes were activated by incubation with alamethicin (50 μg/mg protein) on ice for 15 min as reported previously 10, 38, 39, 40.

Rat intestinal microsomes were incubated in triplicate with 3.4 mm MgCl_2_, 115 μm d‐saccharic acid 1,4 lactone monohydrate, 1.15 mm EDTA and 5 mm uridine 5′‐diphosphoglucuronic acid trisodium salt (UDPGA) for 5 min at 37°C shaken at 900 rpm using a CAT SH10 Heater shaker. The reactions were initiated by the addition of 4‐NP. The final organic solvent (methanol) content was 1% *v*/v. Samples at 2.5, 5, 10 and 20 min were quenched with 1:1 with ice cold acetonitrile containing internal standard AZ1 and stored overnight at −20°C. The following day, 100 μl samples were diluted with 200 μl H_2_O, and spun at 3000 rpm for 15 min, and 200 μl of supernatant taken for analysis. Samples were quantified using a standard curve of 4‐nitrophenyl β‐d‐glucuronide using a LTQ Orbitrap with Accela pump (Thermo Scientific, Waltham, MA, USA) and Synergi MAX‐RP 80 Å, 4 μm, 50 × 2.0 mm (Phenomenex, Torrance, CA, USA) with a guard filter of the same phase.

### Rat intestinal microsome pools

The microsomes prepared from the final optimized preparation method (method 8) were measured for CYP content on the day of preparation prior to storage. These microsomes (*n* = 3 samples prepared on *n* = 3 separate occasions) were subsequently thawed on ice and pooled for pool 1. The pool 1 microsomes were then assayed for testosterone and 4‐NP metabolite formation. Additional intestinal microsomes were created using the identical methodology with the same number of rat intestinal samples (*n* = 3 samples prepared on *n* = 3 occasions) and assayed for testosterone and 4‐NP metabolite formation as well as for CYP content on the day of preparation before storage. The microsomes from this second set of preparations were then thawed and pooled to form pool 2.

### Rat intestinal depletion incubations and correction for nonspecific protein binding

Depletion experiments for the determination of intrinsic clearance (*CL*_int_) for rat intestinal microsome pools 1 and 2 were carried out at 1 mg/ml in 0.1 m phosphate buffer (pH 7.4), except for ipriflavone, nicardipine, saquinavir, tacrolimus, terfenadine and verapamil, which were incubated at 0.5 mg/ml. Midazolam and 7‐hydroxycoumarin were both incubated at 1 mg/ml and 0.5 mg/ml. The microsomes were incubated on ice with alamethacin (50 μg/mg) for 15 min. Microsomes were co‐incubated in duplicate at 37°C with both CYP and UGT cofactors (1 mm NADPH, 3.4 mm MgCl_2_, 115 μm SAL, 1.15 mm EDTA and 5 mm UDPGA as per Kilford *et al*. 39 for 5 min and agitated at 900 rpm using a CAT SH10 heater shaker. Co‐incubation of co‐factors was chosen in order to maximize tissue usage. This method was validated using HW commercial intestinal microsomes by comparing the individual cofactor *CL*_int_ to the combined cofactor *CL*_int_ for eight compounds (Supplemental material Figure 1). Incubations were initiated by the addition of test compound (1 μm, final organic solvent (DMSO) was 1%). The samples were quenched with ice cold acetonitrile (3:1 quench to sample) containing the internal standard (AZ1). The total incubation time was 40 min. Quenched samples were stored overnight at −20°C. The following day, the samples were diluted with 200 μl UPH_2_O, and spun at 3000 rpm (Sorvall legend RT centrifuge) for 15 min, and 200 μl of supernatant taken for analysis.

Nonspecific protein binding was determined in microsomes (1 mg/ml) using high throughput 96‐well micro‐equilibrium dialysis methodology (HTDialysis, LLC, Gales Ferry, CT, USA). These experiments used dialysis membrane strips with 12–14 kDa molecular mass cut‐off (HTDialysis). The microsomes were constituted in 0.1 m phosphate buffer (pH 7.4). The final concentrations of the test compounds was 1 μm. Microsomes containing the test compound were aliquoted into donor wells in triplicate, and buffer placed in the acceptor wells. The plate was incubated at 37°C and left to equilibrate on a plate shaker (450 rpm) for 4 h. Samples from both the acceptor and donor sides of the membrane were then transferred to 96‐well plates and quenched in acetonitrile containing internal standard (AZ1). The same sample preparation and LC–MS/MS methodology was used as described for the microsomal incubations. Compound MS transitions and retention times are reported in Supplemental material Table 3. *F*u_inc_ was calculated as the ratio of acceptor to donor both normalized for internal standard as described previously 41. Correction for protein binding for depletion experiments undertaken at 0.5 mg/ml were made using Equation (4).

(4) $${\mathrm{fu}}_{\mathrm{mic},2}=\frac{1}{\left(\frac{[P]{}_{\mathrm{mic},2}}{{\left[P\right]}_{\mathrm{mic},1}}\times \left(\frac{1-{\mathrm{fu}}_{\mathrm{mic},1}}{{\mathrm{fu}}_{\mathrm{mic},1}}\right)+1\right)}$$

where _mic,1_ denotes 1 mg/ml and _mic,2_ is 0.5 mg/ml.

### In vitro–in vivo extrapolation of rat intestinal intrinsic clearance

The *CL*_int,u_ values were scaled to give *CL*_int,u,gut_ using scaling factors for the corresponding microsomal pool (Eq. (5)). The use of scalars from the mean of both pools was also investigated, and was also used for scaling of commercial microsome *CL*_int,u_ data.

(5) $${\mathrm{CL}}_{\mathrm{int},u,G}={\mathrm{CLu}}_{\mathrm{int}}\cdot \text{MPPGI}\cdot \text{Intestine Segment Weight}$$

### Data analysis

Analyses comparing means using Student's *t*‐test to assess statistical significance at a level of 5% of individual mucosal yields, microsomal CYP content, recovery, and values of corrected and uncorrected microsomal protein per gram of intestine (MPPGI) and microsomal protein per gram of mucosa (MPPGM), for each preparation method were calculated using SPSS Statistics version 20 (IBM, Chicago, IL, USA).

## Results

### Intestinal microsomal preparation optimization

#### Enterocyte elution conditions

**Figure 2:**
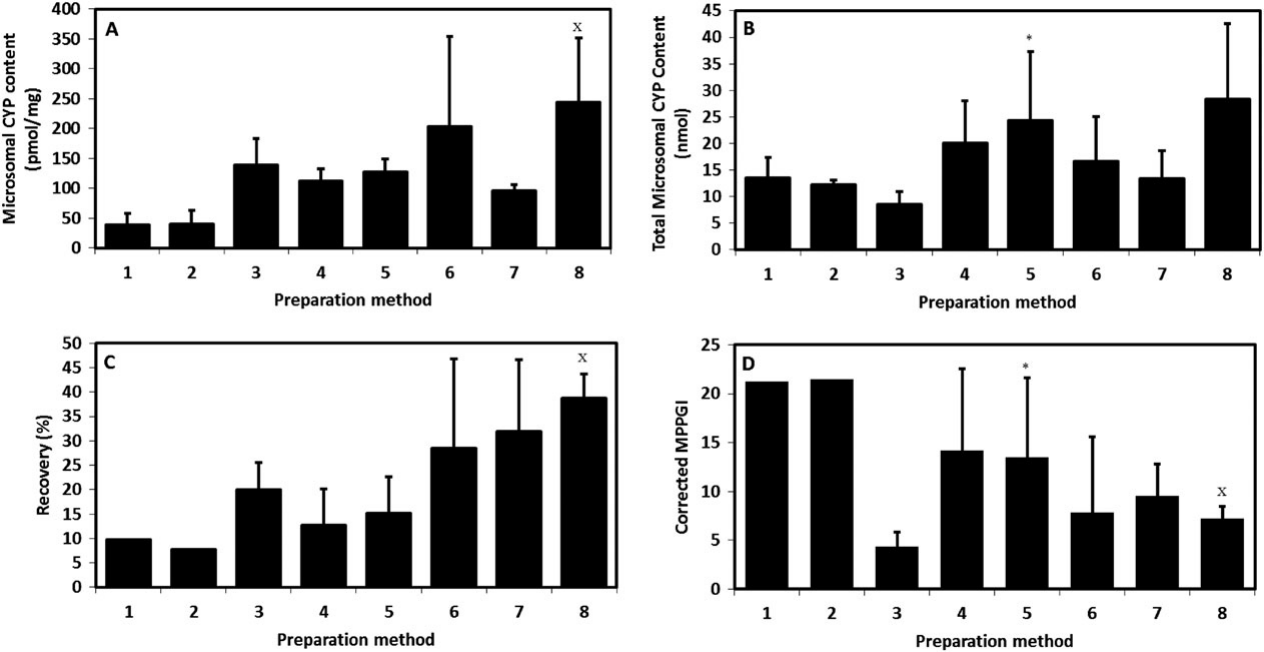
Comparison of microsomal CYP content (A), total microsomal content (B), microsomal recovery (C) and MPPGM (D). *Significantly different to condition (3) (*p* < 0.05), ×Significantly different to condition (5) (*p* < 0.05). Preparation numbers correspond to those in Table 1 and represent the mean ± SD for pooled observations of three rats on three separate occasions. MPPGM, microsomal protein per gram of mucosa

The effect of the preparation methods on recoveries and intestinal scalars is summarized in Table 1 and Figure 2. Comparison of the 20 and 60 min elution procedures undertaken at 5 mm EDTA indicated that incubation times of 20 min produced a significantly lower mean mucosal yield (0.25 vs. 0.47 g/g intestine, *p* < 0.05) (Table 1, Figure 2), but a statistically higher specific microsomal CYP content (138.6 vs*.* 47.6 nmol/mg, *p* < 0.05).

**Table 1:** Summary of intestinal sample weights, mucosal yields, CYP content and recoveries, and respective scalars resulting from differing preparation methodologies

#	Preparation method	Intestinal sample weight^c^ (g)	Mean mucosal yield (g/g intestine)	Uncorrected MPPGI (mg/g)	Uncorrected MPPGM (mg/g)	Recovery (%)	MPPGI (mg/g)	MPPGM (mg/g)	Mean microsomal CYP content (pmol/mg)	Total CYP content (nmol)
1	E1 T1 H1 B1 S1^a^	4.6 ± 0.3	0.47 ± 0.20	2.8	5.9 ± 0.3	9.8	21.2	59.5	39.1 ± 19.2	13.6 ± 3.74
2	E2 T1 H1 B1 S1^a^	4.5 ± 0.4	0.33 ± 0.03	1.6	4.7 ± 0.7	7.7	21.5	64.0	40.7 ± 21.7	12.3 ± 0.84
3	E1 T2 H1 B1 S1	5.0 ± 0.5	0.25 ± 0.01	0.8 ± 0.3	3.4 ± 1.4	19.9 ± 5.7	4.3 ± 1.5	17.7 ± 6.5	138.6 ± 44.1	8.5 ± 2.4
4	E1 T2 H2 B1 S1	4.6 ± 0.3	0.27 ± 0.02	1.4 ± 0.2	5.2 ± 1.7	12.7 ± 7.4	14.2 ± 8.4	51.5 ± 27.4	112.1 ± 21.0	20.1 ± 7.9
5	E1 T2 H3 B1 S1	5.0 ± 0.4	0.22 ± 0.03	1.7 ± 0.3	7.8 ± 0.7	15.1 ± 7.5	13.5 ± 8.2	59.6 ± 27.8	127.0 ± 21.6	24.4 ± 13.0
6	E1 T2 H3 B2 S2^b^	5.4 ± 0.4	0.29 ± 0.10	1.4 ± 0.4	5.2 ± 2.2	28.5 ± 18.2	7.9 ± 7.7	27.9 ± 22.5	203.1 ± 151.4	16.7 ± 8.4
7	E1 T2 H3 B3 S2^b^	4.8 ± 0.5	0.39 ± 0.10	2.8 ± 0.1	7.2 ± 2.0	31.9 ± 14.7	9.5 ± 3.3	24.0 ± 4.1	95.9 ± 10.5	13.3 ± 5.3
8	E1 T2 H3 B4 S1^b^^,^ d	5.4 ± 0.3	0.48 ± 0.02	2.8 ± 0.1	5.8 ± 0.1	38.7 ± 5.0	7.2 ± 1.3	15.1 ± 2.1	243.6 ± 107.7	28.3 ± 14.2

Data represent mean ± SD of *n* = 3 separate preparations of *n* = 3 rats unless indicated.

E1, 5 mm EDTA; E2, 1.5 mm EDTA.

T1, 60 min; T2, 20 min.

H1, 20% (6 W) sonication; H2, 60% (18 W) sonication; H3, 100% (30 W) sonication.

B1, No heparin or glycerol; B2, glycerol 20% *v*/v added to solution C; B3, heparin 3 U/ml and glycerol 20% *v*/v in solution C; B4, heparin 9 U/ml in solution B.

S1, no glycerol; S2, glycerol 20% *v*/v.

a Data derived from *n* = 3 tissues, *n* = 2 homogenate CYP measurements.

b Incubation conditions: 5 mm EDTA, 20 min; homogenization conditions: 100% (30 W) sonication.

c Mean of each individual rat (*n* = 9) corresponds to 60 cm proximal small intestine.

d Also referred to as pool 1. Heparin was present at 3 U/ml in solution B in all preparations except preparations 7 and 8 where it was present at 9 U/ml. MPPGI, microsomal protein per gram of intestine; MPPGM, microsomal protein per gram of mucosa.

For both preparation methods 1 and 2 (60 min incubations) one homogenate sample was excluded from the analysis as the CYP spectrum was around the limit of detection (0.001 absorbance units) for the homogenate samples. Recovery was low in these preparations (Table 1). Incubations of 20 min did yield homogenate with sufficient CYP content to calculate the microsomal recoveries with increased confidence (Table 1, Figure 2). However, the total microsomal protein and CYP yields were still low.

#### Investigations of homogenization intensity

A range of homogenization conditions were investigated and their impact on the CYP content and microsomal yield was assessed for preparations 3–5. No significant change in specific CYP content was observed following homogenization at sonication intensities of 6, 18 or 30 W (138.6, 112.1 and 127.0 nmol/mg, respectively) (Table 1, Figure 2). However, the yield of raw microsomal protein was statistically higher (3.42 vs. 7.77 mg, *p* < 0.05) following treatment at the highest intensity (30 W, preparation method 5) (Table 1). This resulted in a 2.9 fold higher total CYP content of 8.5 vs. 24.4 nmol, *p* < 0.05, for 6 W and 30 W, respectively.

#### Assessment of the heparin and glycerol effect

The effect of the addition of 20% *v*/v glycerol both to the homogenization (solution C) and storage buffers (solution D) (preparation method 6) resulted in a high mean CYP content (203.1 nmol/mg) (Table 1, Figure 2). However, the CV was high (75%). The removal of heparin from solution C, in addition to an increase in heparin content from 3 U/ml to 9 U/ml in solution B, and the removal of glycerol from solutions C and D (preparation method 8) resulted in the highest mean CYP specific content, (243.6 ± 107.7 pmol/mg, *p* < 0.05, CV 44%).

Recovery of microsomal protein was highest with inclusion of glycerol and/or heparin. However, the inclusion of glycerol alone resulted in the highest variability (CV 64%). In comparison with the 100% (30 W) sonication control (preparation method 5) the CV was 50%. In the presence of heparin alone (preparation method 8), the microsomal recovery was 1.9‐fold higher than preparation method 5 with a CV of 12.9%.

#### Characterization of rat intestinal microsomes

The impact of freeze thawing on the preparation methods according to measured specific CYP content is summarized in Table 2. The mean specific CYP content was reduced 39% in freeze thawed (FT) pool 1 vs. the mean of three fresh measurements (Table 2). However, no significant further reduction in CYP content was noted over two additional FT cycles. Similarly, the CYP content was compared after FT in microsomes prepared from frozen intestinal tissue with pool 1 measured from fresh tissue. No further reduction in CYP was observed following further FT cycles. The specific content was 51% lower in pool 2 vs*.* pool 1. Similar CYP levels were observed in a single pool of fresh microsomes from three samples prepared by gentle scraping (115.3 pmol/mg).

**Table 2:** Specific CYP content measured fresh and over three freeze–thaw cycles

Pool	Fresh^a^	FT1^a^	FT2^a^	FT3^a^
Pool 1	243.6 ± 107.7^b^	148.7 ± 28.3	148.5 ± 13.5	136.4 ± 11.3
Pool 2	119.4 ± 75.1^b^	ND	ND	ND
Frozen	127.7 ± 12.9 ^b^	241.5 ± 34.1	166.5 ± 25.0	166.3 ± 43.8
Scraping	115.3	ND	ND	ND

FT, freeze thaw.

a pmol/mg.

b Represents mean results from three separate preparations.

ND, not determined.

The microsomal recovery based on measuring the CYP content in both homogenate and microsomes for each pool is shown in Table 3. The highest recovery was seen in the freshly prepared pools using elution methodology (pools 1 and 2). Although the recovery in pool 1 was highest, it was not significantly different to pool 2. Recoveries in all preparations ranged from 20.8% to 38.7% (CV 13% to 18%). The lowest mean recoveries were observed for microsomes prepared by scraping and from frozen tissue. Frozen tissue yielded the highest mucosal yield (0.66 ± 0.02 g/g intestine). Differences in microsomal scalars were not significant (*p* > 0.2). Corrected microsomal yields based on per gram intestine ranged from 7.2 to 16.4 mg/g intestine. The mean MPPGI for pools 1 and 2, which were freshly prepared by elution, was 9.7 ± 3.6 mg/g intestine.

**Table 3:** Microsomal recoveries and scalars in intestinal microsomal pools and associated maximal rate of formation of major testosterone and 4‐nitrophenol glucuronide metabolite formation in intestinal microsome pools

Pool	State	Intestine sample weight^e^ (g)	Mucosal yield (g/g intestine)	Recovery (%)^a^	MPPGM (mg/g intestine)^a^	MPPGI (mg/g)^a^	6β‐OH TEST (pmol/min/mg)	Androstenedione (pmol/min/mg)	4‐NP Glucuronide (nmol/min/mg)
Pool 1^b^	Fresh FT	5.4 ± 0.3	0.48 ± 0.02	38.7 ± 5.0	15.1 ± 2.1	7.2 ± 1.3	ND	ND	ND
							85.3 ± 32.5	560.7 ± 6.6	70.4 ± 8.9
Pool 2^b^	Fresh FT	5.3 ± 0.2	0.47 ± 0.06	27.5 ± 5.1	25.9 ± 7.4	12.1 ± 3.9	113.3 ± 30.9	429.9 ± 30.9	84.8 ± 28.6
							187.5 ± 77.8	548.7 ± 188.7	71.4 ± 5.9
Frozen^b^^,^ d	FT	5.5 ± 0.5	0.66 ± 0.02	22.4 ± 7.3	20.9 ± 4.5	13.8 ± 2.7	ND	ND	13.0 ± 5.3
Scraping^c^^,^ d	FT	5.0 ± 0.3	0.49	20.8	23.0	11.4	66.1 ± 4.8	457.0 ± 60.4	46.1 ± 1.68
Commercial HW^d^	FT	NA	NA	NA	NA	NA	190 ± 53.5	314.5 ± 55.3	56.1 ± 4.1
Commercial SD^d^	FT	NA	NA	NA	NA	NA	167.2 ± 43.7	371.6 ± 7.2	55.6 ± 4.7

a Based on CYP content in microsomes and homogenates from 60 cm segments from 9 week old male rats.

b Mean of 3 rats on 3 occasions.

c Mean of 3 rats on 1 occasion. e: corresponds to 60 cm proximal small intestine.

d One occasion in triplicate.

Fresh: microsomes analysed on day of preparation before freezing. FT, microsomes analysed following 1 FT cycle; ND, not determined; NA, not available. MPPGI, microsomal protein per gram of intestine; MPPGM, microsomal protein per gram of mucosa.

#### Testosterone metabolite rate of formation in rat intestinal microsomes

The rate of formation of two major testosterone metabolites, 6β‐OH TEST and androstenedione in each of the pools is shown in Table 3. The formation of 16α‐, 16β‐OH TEST was also observed in smaller amounts in rat intestinal microsomes (Supplemental material Table 4). The mean 6β‐ and 16α‐OH TEST formation was 2.2‐ and 2.5‐fold higher in pool 2, respectively. The mean 6β‐OH TEST formation was lowest in scraped microsomes (66.1 pmol/min/mg). The mean 6β‐OH TEST formation was similar for in‐house pools and commercial HW and SD microsomes. However, androstenedione formation was significantly reduced in commercial microsomes. The mean 6β‐OH TEST formation in fresh and FT microsomes (pool 2) were 113.3 and 187.5 pmol/min/mg, respectively.

#### Glucuronidation activity in rat intestinal microsomes

The mean 4‐NP glucuronide formation was the same in both pool 1 and pool 2 following one FT cycle (70.4 and 71.4 nmol/min/min, respectively) (Table 3). The mean activity in the fresh microsomes showed the highest formation rates. However, this was not significant compared with the FT microsomes. Activity in microsomes prepared via scraping was 1.5‐fold lower than pools 1 and 2. The lowest activity was observed in microsomes prepared through the use of frozen intestinal tissue (13.0 nmol/min/mg). Activity in commercial microsomes was similar for both strains and was higher than those prepared via scraping. However, the activities were lower compared with the in‐house pools generated from fresh tissue using the elution technique.

### Rat intestinal microsomal incubations

#### Rat intestinal microsomal binding

The *fu*_inc_ in rat intestinal microsomes for the compounds investigated is shown in Table 4. Microsomal binding at 1 mg/ml ranged from 0% for 7‐hydroxycoumarin, pirenzepine and furosemide to 98% for terfenadine.

**Table 4:** Mean measured protein binding in intestinal microsomal incubations and (*fu*_*i*nc_) and unbound intrinsic clearance *CL*_int,u_ determined from in‐house rat intestinal pools and commercial Han Wistar microsomes using combined and individual CYP and UGT cofactors

Compound	*f*u_inc_ a	*CL*_int,u_ (μl/min/mg)^a^				
		In‐house combined cofactors		Commercial Han Wistar pool		
		Pool 1	Pool 2	Combined cofactors	CYP cofactors	UGT cofactors
7‐Hydroxycoumarin (7‐HC)	1.00	196.5	264.2^a^^,^ b	271.7	0.9	327.3
Amitriptyline (AMT)	0.20	24.3	19.9	23.8	5.3	3.0
Atorvastatin (ATO)	0.57	5.3				
Bisporolol (BIS)	0.89	5.4	< 0.1			
Bumetanide (BUM)	0.92	2.8	7.3			
Buspirone (BUS)	0.91	3.2	1.4			
Cyclosporine A (CYC)	0.82	21.0	30.8	13.7		
Diclofenac (DIC)	0.98	2.6	< 0.1	5.0		
Diltiazem (DIL)	0.85	21.8	26.7			
Furosemide (FUR)	1.00	2.9	< 0.1			
Indomethacin (IND)	0.88	48.9	62.4			
Ipriflavone (IPR)	0.28	495.0	416.2^a^^,^ b	72.0	59.1	7.3
Irbesartan (IRB)	0.79	31.9	35.9	15.2	1.5	9.4
Losartan (LOS)	0.87	34.8	14.8	10.0	0.1	9.0
Midazolam (MDZ)	0.72	22.0	18.2^a^^,^ b	12.3	18.2	2.8
Nicardipine (NIC)	0.09	1780.2	1930.2^b^	865.7	1048.0	1.2
Omeprazole (OMP)	0.90	7.6				
Pirenzepine (PIR)	1.00	3.9	2.7			
Raloxifene (RAL)	0.06	1135.3	1654.3	1042.1	9.8	927.0
Saquinavir (SAQ)	0.11	2198.4^b^	3556.7^b^			
Sildenafil (SIL)	0.73	23.8	18.4			
Simvastatin (SIM)	0.93	13.6	41.5			
Tacrolimus (TAC)	0.32	464.6^b^	413.0^b^			
Terfenadine (TER)	0.02	16264.0^b^	20225.2^b^			
Verapamil (VER)	0.65		25.0^a^^,^ b	6.9		

a Incubations at 1 mg/ml at compound concentration of 1 μm, except for

b which were incubated at 0.5 mg/ml due to high clearance. Predicted protein binding at 0.5 mg/ml from data measured at 1 mg/ml: 7‐hydroxycooumarin (1.00), ipriflavone (0.43), midazolam (0.83), nicardipine (0.17), saquinavir (0.19), tacrolimus (0.48), terfenadine (0.03) and verapamil (0.78).

#### Unbound intrinsic clearance comparison between pools

The measures of unbound clearance corrected for protein binding showed a range of microsomal clearance (Table 4). In pool 1 the microsomal clearance ranged from 2.6 to 16264 μl/min/mg, and in pool 2 from <0.1 to 20225 μl/min/mg, for diclofenac and terfenadine, respectively. The mean *CL*_int,u_ of midazolam was similar in both pools (22.0 and 18.2 μl/min/mg for pool 1 and 2, respectively).

**Figure 3:**
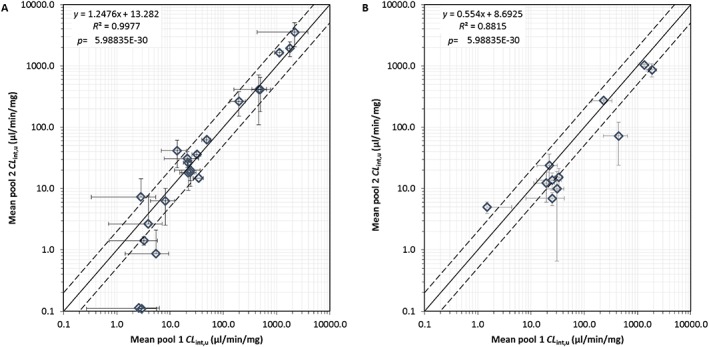
(A) Correlation between *CL*_int,u_ in pool 1 and pool 2 rat intestinal microsomes using combined CYP and UGT cofactors. *n* = 22 compounds. Data represent mean ± SD of *n* = 3 of duplicate incubations. (B) Correlation between *CL*_int,u_ for in‐house pools (1 and 2) and Han Wistar commercial rat intestinal microsomes using combined CYP and UGT cofactors. *n* = 11 compounds. Data represent mean ± SD of *n* = 3 of duplicate incubations. Solid line represents line of unity, dashed lines 2‐fold

The correlation between *CL*_int,u_ values obtained in the two pools of rat intestinal microsomes is shown in Figure 3A. The correlation between the pools was strong (*R* ^2^ = 0.998, *p* < 0.001), with 61% of compounds within 2‐fold. The greatest fold difference between pools was observed for compounds with *CL*_int,u_ below 10 μl/min/mg (diclofenac and furosemide).

#### Comparison of commercial and in‐house intestinal microsomal pools

The mean *CL*_int,u_ for 11 compounds that overlapped with those used in in‐house microsomes were screened in commercial microsomes using combined CYP and UGT cofactors is shown in Table 4. The mean *CL*_int,u_ ranged from 5 to 1042 μl/min/mg based on diclofenac and raloxifene data, respectively. There was a positive correlation between both commercial and in‐house rat intestinal microsomes (*R* ^2^ = 0.88, *p* < 0.001) (Figure 3B). However, 54% of compounds studied showed a greater than 2‐fold higher *CL*_int,u_ using in‐house microsomes. The mean fold difference was 1.7‐fold. Good correlation was observed for midazolam, amitryptiline, 7‐HC and raloxifene.

#### Comparison of combined vs. individual CYP and UGT cofactors

The main route of elimination for midazolam, nicardipine and ipriflavone was via CYP mediated metabolism, whereas glucuronidation was the major clearance pathway for 7‐HC, raloxifene, irbesartan and losartan. The contribution of both CYP and UGT metabolism was comparable only in the case of amitryptiline.

When comparing the mean *CL*_int,u_ obtained in the presence of combined cofactors and additive *CL*_int,u_ for individual cofactors, 2‐fold for all compounds, with the exception of amitryptiline, and a strong positive correlation was observed (*R* ^2^ = 0.966, *p* < 0.001) (Table 4, Supplementary material Figure 1). The mean CV was lowest for individual cofactors (39% *vs.* 58% for individual and combined cofactors, respectively).

### In vitro–in vivo extrapolation of rat intestinal intrinsic clearance

**Figure 4:**
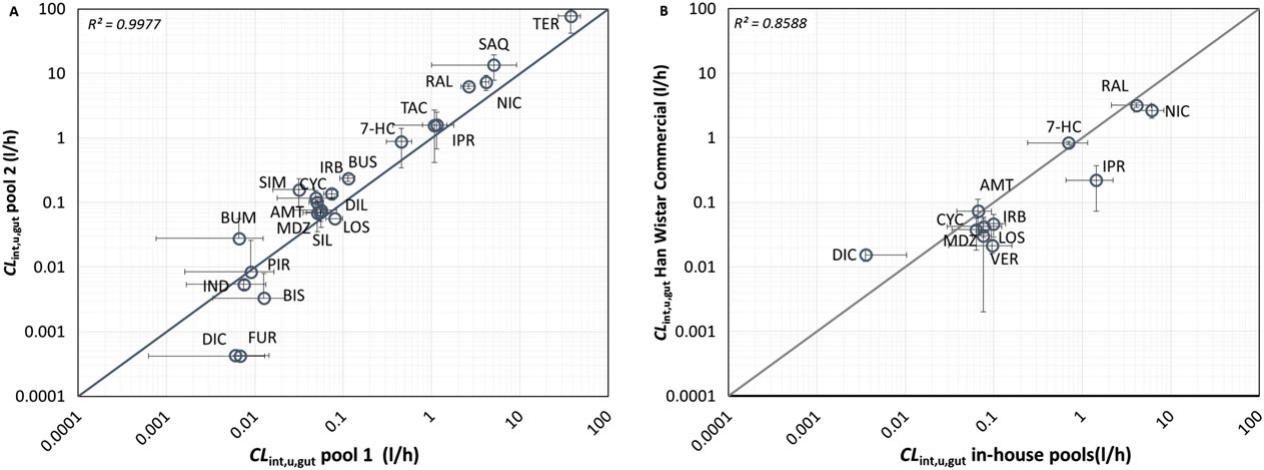
Comparison of extrapolated measures of *CL*_int,u,gut_ (l/h) for individual pools and commercial microsomes for a range of study compounds. (A) Comparison of pool 1 vs*.* pool 2 extrapolated *CL*_int,u,gut_. Line of unity (solid line). Extrapolated *CL*_int,u,gut_ (l/h) for pool 1, pool 2 were scaled using pool specific scaling factors of intestinal weight and microsomal protein per gram intestine. (B) Comparison of in‐house and commercial microsomes extrapolated *CL*_int,u,gut_. Line of unity (solid line). In‐house pooled and Han Wistar commercial microsomes were scaled using mean weights and scalars were used from the two in‐house prepared pools. Compound abbreviations in Table 5

**Figure 5:**
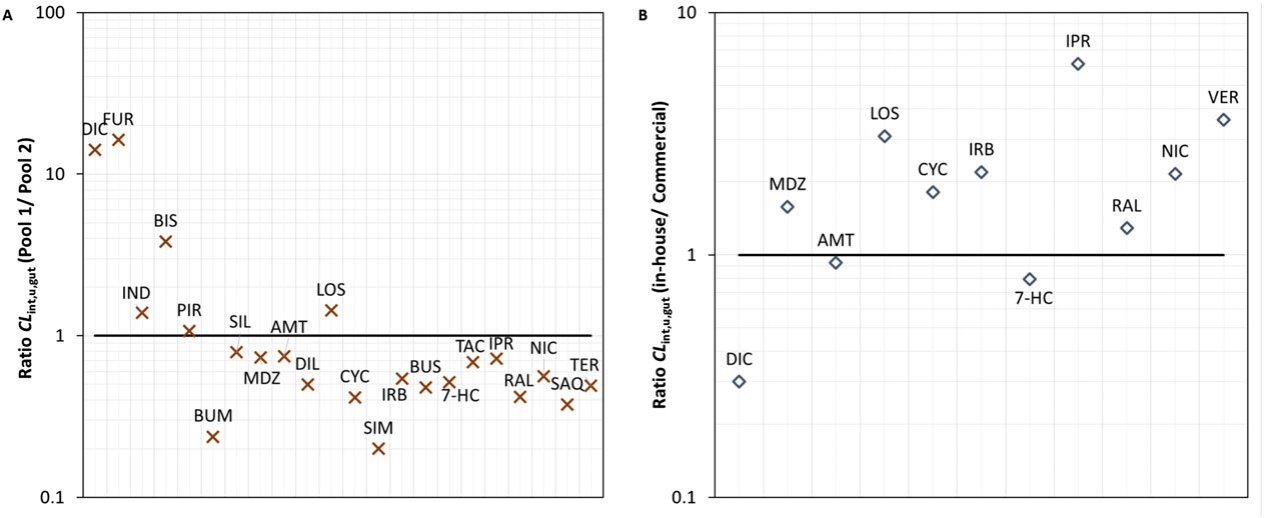
Ratios of extrapolated measures of *CL*_int,u,gut_ (l/h) for individual pools and comparison with commercial microsomes for a range of study compounds. (A) Ratio of pool 1 and pool 2 extrapolated *CL*_int,u,gut_. Line of unity (solid line). Extrapolated *CL*_int,u,gut_ (l/h) for pool 1, pool 2 were scaled using pool specific scaling factors of intestinal weight and microsomal protein per gram intestine. (B) Ratio of in‐house and commercial microsomes extrapolated *CL*_int,u,gut_. Line of unity (solid line). In‐house pooled and Han Wistar commercial microsomes were scaled using mean weights and scalars were used from the two in‐house prepared pools. Compound abbreviations in Table 5

The range of extrapolated *in vitro CL*_int,u,gut_ for both individual in‐house pools and Han Wistar commercial microsomes is shown in Table 5 and Figure 4. The individual compound predicted *CL*_int,u,gut_ was similar between separate pools using the pool specific scaling factors (*R* ^2^ = 0.9977). The fold difference in the ratio of *CL*_int,u,gut_ ranged from 0.2‐ to 16‐fold for simvastatin and furosemide, respectively. The mean fold difference and the geometric mean fold error between pools of 2.1 and 1.2‐fold, respectively for *n* = 22 compounds (Figure 5A).

**Table 5:** Extrapolated intestinal unbound intrinsic clearance (*CL*_int,u,gut_) from rat intestinal microsome pools

Compound	*CL*_int,u,gut_ (l/h)			
	Pool 1 Mean	Pool 2 Mean	Combined in‐house pools	Mean Han Wistar commercial microsomes
7‐Hydroxycoumarin (7‐HC)	0.06	0.08	0.07	0.07
Amitriptyline (AMT)	0.01	NM	0.01	
Atorvastatin (ATO)	0.01	< 0.01	0.01	
Bisporolol (BIS)	0.01	0.03	0.02	
Bumetanide (BUM)	0.11	0.24	0.16	
Buspirone (BUS)	0.05	0.12	0.08	0.04
Cyclosporine A (CYC)	0.01	< 0.01	< 0.01	0.02
Diclofenac (DIC)	0.05	0.10	0.07	
Diltiazem (DIL)	0.01	< 0.01	< 0.01	
Furosemide (FUR)	0.01	0.01	0.01	
Indomethacin (IND)	1.15	1.59	1.44	0.22
Ipriflavone (IPR)	0.07	0.14	0.10	0.05
Irbesartan (IRB)	0.08	0.06	0.08	0.03
Losartan (LOS)	0.05	0.07	0.06	0.04
Midazolam (MDZ)	4.13	7.36	6.12	2.66
Nicardipine (NIC)	NM	0.14	0.14	
Omeprazole (OMP)	0.01	0.01	0.01	
Pirenzepine (PIR)	2.64	6.31	4.10	3.21
Raloxifene (RAL)	5.09	13.56	10.17	
Saquinavir (SAQ)	0.06	0.07	0.06	
Sildenafil (SIL)	0.03	0.16	0.08	
Simvastatin (SIM)	1.08	1.57	1.38	
Tacrolimus (TAC)	37.77	77.09	59.22	
Terfenadine (TER)	NM	0.10	0.10	0.02

A threshold of 0.01 l/h was assumed for *in vitro CL*_int,u,gut._ NM, not measured.

Extrapolations were also made using the mean of pool 1 and pool 2 scaling factors applied to the mean *CL*_int,u_ derived from both in‐house pools and commercial microsomes (Table 5). The ratio for a smaller set of compounds (*n* = 11) comparing extrapolations from measured *CL*_int,u_ from the mean of in‐house pools and Han Wistar commercial microsomes, is shown in Figure 5B. The fold difference in the ratio of *CL*_int,u,gut_ ranged from 0.3‐ to 6.1‐fold for diclofenac and ipriflavone, respectively. The mean fold difference and the geometric mean fold error between the pools were 2.2 and 1.7‐fold, respectively.

## Discussion

Intestinal microsomes have been used previously to investigate intestinal metabolism, however, questions have remained about the optimal preparation methods and have been limited in their metabolic potential, leading to poor confidence in estimating the impact of intestinal metabolism 9, 12. With this in mind, key steps of the microsomal preparation were assessed.

Most commonly it is accepted that elution using EDTA is a preferred method for intestinal microsome preparation due to the increased activity 42, 43, a presumed result of lower contamination and damage to the CYP enzymes, as well as improved reproducibility 20, 44. EDTA binding to calcium disrupts the joining of cadherins responsible for cell adhesion, and allows cell detachment 24. Two commonly applied EDTA concentrations of 1.5 mm and 5 mm were chosen for comparison 19, 20. The mean mucosal yields were greatest at 5 mm EDTA and with a longer incubation (60 min (preparation method 1) and also when the heparin content was higher in solution B (preparation method 8) using a 20 min incubation (0.47 and 0.48 g/g intestine, respectively). However, the mean specific microsomal CYP content was 5.3‐fold lower in preparation method 1 relative to 8 (Table 1).

When measured in the intestinal homogenate using a 60 min incubation time, the CYP content was around the limit of detection meaning no reliable estimate of recoveries could be made, and therefore microsomal scaling factors could not be reliably estimated. Due to the heterogeneous expression of cell types within the intestinal mucosa (for example mucus‐secreting goblet cells, endocrine cells and Paneth cells), the enterocytes only account for approximately 25% of the total wet weight 26, 45, 46. Therefore, a shorter incubation time (20 min) was selected to ensure minimal contamination by non‐enterocytic cell types; in turn improving the measurement of CYP content for more reliable estimation of CYP recovery. As a result, a statistically lower mucosal (i.e. enterocyte) wet weight yield (1.9‐fold), and a higher microsomal CYP content (138.6 nmol/mg *vs.* 40.7 nmol/mg, *p* < 0.05) was observed, suggesting a reduced contamination. Furthermore, it is likely that a reduced preparation time would improve the CYP yield by reducing CYP damage 26. Using the spectroscopy method employed it was not possible to quantify whether the higher CYP yield at 20 min was a result of a lower P420 formation, characteristic of P450 inactivation 27.

The application of sonication to promote increased release of microsomal protein is necessary to ensure complete disruption of enterocytes 47. However, CYP enzymes are sensitive to the intensity of homogenization. This has been demonstrated with liver microsomes where 7‐ethoxycoumarin O‐deethylase activity decreased following treatment at 30 W for over 20 s 48. Given these sensitivities, it was important to ensure that increasing the homogenization intensities did not negatively impact the intestinal microsomal preparation. Using increasing homogenization intensities (6, 18, 30 W, all two sets of 10 s bursts) no significant change in CYP content was observed. However, the microsomal yields were statistically higher (approximately 2‐fold) and in agreement with a 1.5‐fold increase in the total CYP yield. This suggested the homogenization intensities caused minimal damage to the CYP enzymes, whilst increasing the overall microsomal yields, given the mean microsomal content was relatively unchanged (1389 ± 44.1 *vs*. 127 ± 21.0 for preparation methods 3 and 5, respectively).

Glycerol is routinely added to homogenization buffers during the preparation of hepatic microsomes. Its addition has been reported to protect CYP enzymes during homogenization procedures and has been applied successfully for the preparation of other extrahepatic microsomes e.g. lung 26. The mean specific CYP content in the absence of glycerol was 36% lower than when it was present in the homogenization buffer (solution C, preparation 6). This was in line with previously reported data which suggested a 25–35% reduction when glycerol was excluded 27. However, the inclusion of glycerol was poorly reproducible since the CV increased compared with the no glycerol control (44% *vs.* 75%), and there was no significant difference in the total CYP protein (8.8 ± 0.7 nmol *vs.* 16.7 ± 8.4 nmol for preparation methods 5 and 6, respectively). Therefore, the inclusion of glycerol was deemed not to be beneficial to intestinal microsomal preparation considering its poor reproducibility, in agreement with recent reports 16. Alternative strategies, including the addition of heparin were therefore investigated to improve microsome preparation.

Intestinal mucus represents a particular problem to the preparation of microsomal fractions since it tends to aggregate cellular and subcellular material thereby influencing the homogeneity of the preparations 49, 50. Strategies to limit mucus contamination have predominantly focused on flushing the intestine prior to enterocyte isolation. Preparation of enterocytes via elution partially limits mucus contamination compared with scraping techniques. This is thought to be due to the more selective isolation of the epithelial layer from the underlying lamina propria 18. However, the prevention of mucus contamination is not complete. Although the addition of heparin prevents agglutination and protein aggregation, and has been reported to increase the protein yield up to 30%, it comes at the expense of decreasing the CYP concentration 26, 27.

Indeed the addition of heparin to solution C had a dramatic effect on both microsomal protein yields and specific CYP contents. When heparin and glycerol were both present and compared with the ‘no addition’ control (preparation method 5) there was a 31% reduction in specific CYP content and a 3.3‐fold increase in the raw microsomal protein yield. To the best of our knowledge no‐one has published the effect of heparin alone at 9 U/ml in solution B. However, the inclusion of heparin resulted in a 1.8‐fold increase in the CYP specific content. The measured values were similar to those reported in rat intestinal microsomes prepared from the Sprague Dawley rat (0.23 ± 0.04 nmol/mg) 16. Furthermore, microsomal protein was also higher, resulting in a 1.7‐fold increase in total CYP content, the highest from all of the conditions investigated.

### Impact of preparation conditions on recovery and correction for losses

To date, corrected scaling factors for the rat intestine have not been reported in the literature. However, estimates of microsomal recovery in rat intestine, measured through aryl‐esterase activity, have been reported at between 45% and 60% 18, 27. Mean recovery in this investigation, in the absence of heparin and/or glycerol, ranged between 12.7% and 19.8%. The inclusion of glycerol increased recoveries to 28.5%–31.9%. However, the highest recovery (38.7%) was observed with preparation method 8, where 9 U/ml heparin was present in solution B during the elution step. This resulted in the lowest CV (13%) and corresponded to a 2.2‐fold increase in mucosal yield, suggesting that heparin provided a greater wet weight of enterocytes (most likely through preventing aggregation of protein by mucus). The total mean mucosal yields, expressed per intestine, using this method were 0.48 g/g intestine, similar to those reported previously (0.33 g/intestine) 9, 18, 19, 51. The largest yield of raw microsomal protein was also observed under these conditions suggesting that the higher heparin content in the initial elution also promoted higher microsomal yields; most probably due to lower mucus contamination.

The impact of homogenization intensity had the greatest effect on microsomal scalars. Comparing 20% (6 W) and 100% (30 W) homogenizations, there was a significant (2‐fold) increase for all normalized scalars. However, when comparing all the homogenizations at 30 W, a significant difference was noted between preparation methods 5 and 8 only when expressed per gram of mucosa. In this case, a significantly lower (3.9‐fold) scalar was obtained for method 8 vs. method 5 (expressed per gram mucosa). By comparison, the fold difference in MPPGI was 1.7‐fold. Interestingly, the fold difference between MPPGM and MPPGI for preparation 8 was 2.2‐fold, reflecting the observed fold difference in mucosal yields between both preparations. These findings highlight the sensitivity of this scalar to the increased wet weight of mucosal cells yielded with the addition of 9 U/ml heparin in solution B.

To the best of the authors' knowledge, the current study represents the first systematic report that assesses the impact of different preparation conditions of intestinal microsomes on corrected microsomal scalars within the same laboratory. An optimized method for microsomal preparation was devised. As demonstrated, the technique employed for enterocyte preparation and intestinal microsomal preparation influences both the specific and total enzyme content, as well as the microsomal protein yield. Unlike MMPGM expressed scalars, no significant differences in corrected scalars of MPPGI were observed for all of the 100% (30 W) sonication preparations (preparation methods 5–8). This indicates that this scalar was sensitive to the wet weight of mucosa, and suggests the impact of preparation methods on the value of intestine scaling factor. Therefore, when comparing between laboratories, a normalization based on intestinal weight or length may be more comparative, whereas measures based on a per gram mucosa basis may lead to increased discrepancies, unless mucosal yields are also provided. Furthermore, the impact of method on the scalar highlights the importance of determining the scalar for a specific preparation method; as this is likely to impact the prediction success of *in vitro–in vivo* extrapolation (IVIVE).

Two pools of microsomes (pool 1 and pool 2) were prepared using identical conditions in order to determine the reproducibility of the isolation methodology, and to characterize the drug metabolizing enzyme activity using a range of substrates. Although a lower recovery and higher scalar were observed in pool 2 vs. pool 1, these results were not statistically significant.

Intestinal microsomes were also prepared using frozen tissue samples. However, the use of frozen rat intestinal tissue resulted in a larger enterocyte yield. When flushed, unlike fresh tissue, the intestine was easily stripped of its mucosa. This may in part be due to the simple tube‐like structure of the rat intestine which, unlike human tissue, is not folded (absent of plicae circulares) 17, 52. Finally, on one occasion, microsomes were prepared using a gentle scraping technique. Interestingly, a similar enterocyte yield and corrected microsomal scalars could be achieved through scraping intestinal tissue gently using a glass microscope slide. In general these scraped microsomes showed a lower CYP content as well as a lower mean CYP and UGT activities in line with observations from previous observations 42. However, this was not to the same extreme where virtually no CYP activity and a 50% reduction in *p*‐nitrophenol activity was observed in scraped vs. elution prepared microsomes was reported 42. Although this was only a single preparation, this suggests that given adequate care in preparation, a reduced detrimental impact of scraping can be mitigated.

The mean CYP content was 2‐fold lower in pool 2 vs. pool 1 when measured in fresh preparations. The impact of freeze thawing for pool 1 showed a 39% reduction in CYP content following one FT cycle. Subsequent FT cycles showed no further decrease suggesting that the CYP was stable following the initial freezing. Interestingly, in frozen tissue, when microsomes were measured on the day of preparation the CYP levels were similar across the FT samples, suggesting that any reduction of CYP occurred following the first freezing of either tissue or microsomes.

A fall in the specific CYP content following freezing was not matched by any significant reductions in testosterone metabolite formations in pool 2. The main metabolites observed were 6β‐, 16α‐, 16β‐OH TEST and androstenedione; representing the activities of cyp3a1, and cyp2b 33. No peaks were observed for 2α or 6α suggesting that minimal cyp2c11 or cyp2a1 content was present in Wistar rats. In Wistar rats, cyp2c11 protein expression has not been reported 22 and cyp2a1 has not been detected at an mRNA level 21, This differed with observations in Sprague–Dawley rats where high levels of the 6α‐OH TEST metabolite have been reported 33. Interestingly in commercial Sprague–Dawley microsomes, the formation of 2α‐OH TEST or 6α‐OH TEST were not observed. Conversely levels of 16α‐OH TEST were highest, possibly indicating a role of cyp2c11. However, cyp2b has been identified as a major enzyme expressed in Wistar rat intestines, accounting for the majority of CYP protein expression 20, 21, 22. Androstenedione, a substrate for cyp2b, was also the major metabolite observed in all microsomes studied; suggesting cyp3a is not the dominant route of metabolism in the rat intestine. It should be noted that in addition to contribution of cyp2b, androstenedione formation is also be mediated by 17 β‐hydroxysteroid dehydrogenase.

The formation of 6β‐OH TEST in Wistar rat intestinal microsomes prepared by elution microsomes was slightly lower compared with previously reported values (289 ± 5 pmol/min/mg) 42. However, this is likely due to the shorter length of intestine used in the previous analyses (30 cm) and the fact that cyp3a content has been reported to be highest in the proximal regions and to decrease along the length of the rat small intestine 21, 22. In line with previous reports using microsomes prepared through scraping of the intestinal tissue, the lowest activity in CYP3A (6β‐OH TEST formation) was observed 42, 43.

The rate of 4‐NP glucuronidation was also low in microsomes prepared through scraping compared with elution (using fresh tissue). No reduction in activity was observed in glucuronidation activities using fresh or FT microsomes. Glucuronidation was, however, 10‐fold higher than previously reported (7 ± 0.81 nmol/min/mg) 42. Glucuronidation was lowest in microsomes prepared from frozen tissue. This may be a result of the higher mucosal tissue yield in the initial enterocyte elution procedure resulting in a dilution of active enzyme, however, this was not determined for CYP metabolism.

### Rat intestinal pool depletion experiments

Compounds were selected for screening on the basis that they represented a diverse range of metabolic pathways and intestinal metabolic stabilities. When comparing rat intestinal pools, similar measures of unbound intrinsic clearance for the same set of compounds, and a strong correlation, were observed between the pools (*R* ^2^ = 0.998, *p* < 0.001), with 61% of compounds being within 2‐fold. The weakest correlation was observed for compounds with a *CL*_int,u_ < 10 μl/min/mg. This likely represents the reduced sensitivity of the parent depletion method for measurement of low rates of metabolism (requirement for at least 20% of substrate metabolism) 53.

A positive but weaker correlation (*R* ^2^ = 0.88 *p* < 0.001) was observed between the mean *CL*_int,u_ determined from pooled vs*.* Wistar commercial microsomes. This was in line with higher activity in the in‐house microsomal pools compared with a previous study using commercially available Sprague–Dawley microsomes where metabolism was negligible for the majority of compounds studied 12. However, the measured *CL*_int,u_ (correcting for *fu*_inc_ in this study) showed similar activity for cyp3a substrate midazolam (12.3 vs. 8.3 μl/min/mg). A higher activity for nicardipine (865.7 *vs.* 300 μl/min/mg) was observed with the Wistar commercial microsomes used in this study 12. Since the cyp3a activities determined using 6β‐OH‐TEST formation were observed to be similar in Wistar and SD microsomes used in this study, the higher activity for nicardipine may be related to other metabolism pathways in the Wistar rat.

Of the 11 compounds studied here, a good correlation was observed for midazolam, amitriptyline, 7‐HC and raloxifene. Higher metabolism using in‐house pools was observed for six compounds. Of these compounds, two undergo cyp1a2 metabolism to some extent (pirenzepine and ipriflavone). It was not possible to determine whether this was cyp1a2 related, given that none of the testosterone metabolites are selective for this rat isoform.

Microsomal yields were low from intestinal tissue, and as a way of maximizing their potential for screening of compounds, a combination of CYP and UGT cofactors were utilized in the *CL*_int_ experiments. In order to validate the use of combined cofactors, these were compared with the individual CYP and UGT cofactors as per Kilford *et al.* 39, in the absence of 2% bovine serum albumin. Eight compounds were screened in commercial microsomes using either individual cofactor or combined cofactor incubations. The additive *CL*_int,u_ were strongly correlated (*R* ^2^ = 0.966, *p* < 0.001), with only amitryptyline outside 2‐fold range, suggesting no limitations to the use of combined cofactors in intestinal microsomes, as observed previously using liver microsomes 39.

### In vitro–in vivo extrapolation of rat intestinal intrinsic clearance

The extrapolated *CL*_int,u,gut_ using pool specific scaling factors and measures of intrinsic clearance were comparable for in‐house pools across the 25 compounds studied. For the 22 compounds that overlapped between the two in‐house pools, a strong positive correlation (*R* ^2^ = 0.9977) was observed. The largest fold error was seen for drugs (e.g. diclofenac, 14‐fold and furosemide, 16‐fold) with a low *CL*_int,u,gut_ (less than 0.01 l/h). Considering assay sensitivity, it is reasonable to exclude drugs with such *CL*_int,u,gut_ values and the expected contribution of intestinal metabolism. Using a *CL*_int,u,gut_ of 0.01 l/h as a cut‐off, the mean fold difference was 0.67 between pools for the remaining 18 compounds.

Extrapolation of data obtained in Han Wistar commercial microsomes in general resulted in a lower *CL*_int,u,gut_ compared with the in‐house microsomes, in agreement with the measured intrinsic clearance. On average a 2.2‐fold lower clearance was seen in the commercial microsomes across the 11 compounds studied, with the largest fold difference observed for ipriflavone, as discussed previously.

Various mathematical models have been described for the prediction of *F*_G_ *in vivo*. The *Q*_gut_ model incorporates both permeability and intestinal villous blood flow in addition to measures of intestinal intrinsic clearance 54, 55. Although widely used, it is recognized that this model has limitations; for example improved predictions are observed using the assumption that the fraction unbound in the gut is negligible (*fu*_gut_ = 1) 11, 55, and poor predictions are observed when compounds demonstrate high protein binding and/or are substrates for intestinal transporters (e.g. P‐gp) 11.

Furthermore, the prediction success of intestinal metabolism is impacted by the ability to adequately define the extent of *F*_G_ *in vivo*. Generally, estimates of the *in vivo* contribution of the intestine to oral bioavailability can be made by using indirect measures of i.v. clearance and oral bioavailability 5, 11, ensuring low doses are selected in order to minimize the saturation of intestinal enzymes 4. However, there are several assumptions: for example negligible metabolism occurring in enterocytes after i.v. administration and that systemic clearance of a drug after i.v. dose (corrected for renal excretion) reflects only hepatic elimination. The validity of this assessment is questionable, as for certain drugs the enterocytic contribution has been observed following i.v. administration, e.g. midazolam 56. Furthermore, using this indirect method, estimates of intestinal metabolism are sensitive to the value of hepatic blood flow used, as discussed previously 56. Predictions of *F*_G_ using the prepared microsomes and the assessment of the preparation method on *in vitro–in vivo* extrapolation success remains an important area warranting further investigation.

## Conclusions

The impact of variations to elution conditions, homogenization intensities and the effect of heparin and glycerol on the preparation of intestinal microsomes were investigated. A shorter incubation time using 5 mm EDTA and 9 U/ml heparin, and 30 W sonication, were preferential and resulted in a higher CYP specific and total protein content in the proximal regions of rat intestine. The sensitivity of microsomal yield to preparation methods highlights the impact on IVIVE strategies and the requirement to define the intestinal scalar relevant to the preparation method used. Intestinal rat microsomes prepared by the optimized method showed good reproducibility and metabolic activity using a validation dataset of 25 CYP and UGT substrates. Testosterone hydroxylation highlighted major enzyme pathways of cyp2b and cyp3a in the Wistar rat. Metabolism of 4‐NP as well as other UGT substrates identified glucuronidation as an important elimination pathway in the rat intestine. The validity of combined cofactor conditions for the screening of dual CYP and UGT probes in intestinal microsomes was confirmed.

*In vitro–in vivo* extrapolation of measured intrinsic clearance from intestinal microsomes using the obtained scaling factors showed good agreement between the pools prepared from optimized methods. Agreement to data obtained in commercial microsomes was seen, although trend of lower metabolic activity was evident for the current pools. The impact of these differences on the quantitative prediction of *F*_G_ remains an area of further research, together with investigation of intestinal metabolism in other preclinical species and human.

## Conflict of Interest

The authors declare no conflict of interest

## Supporting information


**Supplemental Figure 1.** Correlation between *CL*_int,u_ for commercial HW rat intestinal microsomes using combined and individual CYP and UGT cofactor incubations. *n* = 8 compounds. Data represent mean ± SD of *n* = 3 of duplicate incubations. Solid line represents line of unity, dashed lines 2‐fold.

**Supplemental Table 1.** Testosterone metabolites and respective rat cyp isoform.
**Supplemental Table 2.** Testosterone hydroxy metabolite elution times and LLOQ.
**Supplemental Table 3.** MS transitions for compounds in depletion studies in RIM, DIM, DLM and HIM, and pharmacokinetic studies in rat and dog blood and plasma.
**Supplemental Table 4.** Maximal rate of formation of testosterone hydroxylation and 4‐nitrophenol glucuronide metabolites in intestinal microsome pools.
