# Supplementary material for: Optimization of intestinal microsomal preparation in the rat: A systematic approach to assess the influence of various methodologies on metabolic activity and scaling factors
Source: Biopharm Drug Dispos. 2017 Apr 18;38(3):187–208. doi: 10.1002/bdd.2070 (PMC5413848; doi:10.1002/bdd.2070)
Supplement: Supplementary file 1 — Supplemental Figure 1. Correlation between CL int,u for commercial HW rat intestinal microsomes using combined and individual CYP and UGT cofactor incubations. n = 8 compounds. Data represent mean ± SD of n = 3 of duplicate incubations. Solid line represents line of unity, dashed lines 2‐fold. [file BDD-38-187-s002.tif]

**Supplemental Figure Legends**

**Supplemental Figure 1 Correlation between CL_int,u_ for commercial HW rat intestinal microsomes using combined and individual CYP and UGT cofactor incubations.** n=8 compounds Data represent mean ±std of n=3 of duplicate incubations. Solid line represents line of unity, dashed lines 2 fold.

**Supplemental material Table 1 Testosterone metabolites and respective rat cyp isoform**

| **Metabolite** | **cyp Isoform** |
| --- | --- |
| 2α-OHTEST | 2c11 |
| 2β-OHTEST | 3a1, 1a1, 1a2, |
| 6α-OHTEST | 2a1 |
| 6β-OHTEST | 85% specific to 3a1 (1a1, 1a2, 3a1) |
| 7α-OHTEST | 2a1 |
| 15α-OHTEST | 2c13, 2a2 |
| 15β-OHTEST | 3a1 |
| 16α-OHTEST | 2c11, 2b1, 2b2, 2c13 |
| 16β-OHTEST | 2b1 |
| Androstenedione* | 2c11,2b2, 2b1 |

Reported from: Sohlenius-Sternbeck, A.K. and A. Orzechowski, Characterization of the rates of testosterone metabolism to various products and of glutathione transferase and sulfotransferase activities in rat intestine and comparison to the corresponding hepatic and renal drug-metabolizing enzymes. Chem Biol Interact, 2004. 148(1-2): p. 49-56. *Androstenedione formation is also be mediated by 17 beta hydroxysteroid dehydrogenase.

**Supplemental material Table 2 Testosterone hydroxy metabolite elution times and LLOQ**

| **Metabolite** | **MW** | **MS Mass (Da)** | **LLOQ (pmol/ml) Method A** | **LLOQ (pmol/ml) Method B** | **Mean Retention Time (min) Method A** | **Mean Retention Time (min) Method B** | **Internal Standard** |
| --- | --- | --- | --- | --- | --- | --- | --- |
| 2-α | 304 | 305 | 100 | 50 | 5.42 | 5.31 | 11-beta |
| 2-β | 304 | 305 | 100 | 50 | 5.80 | 5.55 | 11-beta |
| 6- α | 304 | 305 | 100 | 50 | 2.48 | 2.71 | 11-beta |
| 6- β | 304 | 305 | 100 | 100 | 3.05 | 3.21 | 11-beta |
| 7- α | 304 | 305 | 100 | 100 | 3.45 | 3.63 | 11-beta |
| 11- α | 304 | 305 | 100 | 50 | 5.21 | 4.73 | 11-beta |
| 11- β | 304 | 305 | - | - | 4.78 | 5.21 | - |
| 15- α | 304 | 305 | 100 | 100 | 3.18 | 3.44 | 11-beta |
| 15- β | 304 | 305 | 100 | 50 | 2.55 | 2.83 | 11-beta |
| 16- α | 304 | 305 | 100 | 100 | 4.18 | 4.27 | 11-beta |
| 16- β | 304 | 305 | 100 | 50 | 4.48 | 4.58 | 11-beta |
| Androstenedione | 286 | 287 | 100 | 50 | 6.32 | 6.63 | 11-beta |

LLOQ: Lower Limit of quantification

**Supplemental material Table 3 MS transitions for compounds in depletion studies in RIM, DIM, DLM and HIM, and pharmacokinetic studies in rat and dog blood and plasma.**

| **Compound** | **Ion Class** | **MW (Da)** | **Transition (Da)** | **Col Voltage (V)** | **Col. Energy** | **Retention Time (min)** |
| --- | --- | --- | --- | --- | --- | --- |
| Internal Standard AZ1 | Base | 407.48 | 408.18>207.54 | 35 | 22 | 1.33 |
| 7-Hydroxycoumarin | Neutral | 162.14 | 163.04 > 107.05 | 35 | 10 | 1.22 |
| Amitryptiline | Base | 277.41 | 278.19 > 91.23 | 40 | 22 | 1.77 |
| Atorvastatin | Acid | 558.65 | 559.26 > 440.60 | 25 | 22 | 1.71 |
| Bisporolol Fumerate | Base | 325.45 | 326.23 > 116.03 | 35 | 10 | 1.28 |
| Bumetamide | Acid | 364.42 | 365.12 > 184.17 | 40 | 22 | 1.00 |
| Buspirone | Base | 385.51 | 386.26 > 122.02 | 35 | 22 | 1.26 |
| Cimetidine | Base | 252.34 | 253.22 > 159.66 | 55 | 12 | 0.94 |
| Cyclosporine A | Neutral | 1202.62 | 1204.08 > 1206.50 | 35 | 22 | 1.51 |
| Diclofenac | Acid | 296.15 | 294.00 > 250.00 | 40 | 10 | 1.57 |
| Diltiazem | Base | 414.52 | 415.17 > 177.99 | 35 | 22 | 1.33 |
| Domperidone | Base | 425.91 | 426.17 > 175.06 | 35 | 22 | 1.28 |
| Felodipine | Base | 384.25 | 385.23 > 339.83 | 35 | 12 | 1.46 |
| Furosemide^a^ | Acid | 330.75 | 332.07 > 90.90 | 35 | 25 | 1.47 |
| Indomethacin | Acid | 357.79 | 358.09>139.91 | 35 | 22 | 1.47 |
| Ipriflavone | Neutral | 280.32 | 281.22 > 239.66 | 35 | 12 | 1.48 |
| Irbesartan | Zwitterion | 428.54 | 429.24 > 207.01 | 35 | 22 | 1.33 |
| Losartan K | Acid | 422.92 | 424.35 > 406.96 | 35 | 12 | 1.4 |
| Midazolam HCl | Neutral | 325.77 | 326.50 > 291.50 | 35 | 22 | 1.36 |
| Nicardipine | Base | 479.53 | 480.21 > 315.21 | 35 | 22 | 1.34 |
| Nitrendipine | Base | 360.13 | 361.35 > 315.74 | 55 | 12 | 1.44 |
| Omeprazole | Neutral | 345.42 | 346.25 > 182.04 | 35 | 12 | 1.2 |
| Pirenzepine HCl | Base | 351.41 | 352.18 > 113.11 | 35 | 22 | 1.33 |
| Raloxifene HCl | Base | 473.59 | 474.35 > 112.71 | 95 | 36 | 1.31 |
| Saquinavir Mesylate | Base | 670.85 | 671.39 > 570.42 | 35 | 34 | 1.4 |
| Sildenafil | Base | 474.58 | 475.21 > 100.08 | 35 | 22 | 1.33 |
| Simvastatin | Neutral | 418.57 | 419.28 > 199.06 | 35 | 10 | 1.49 |
| Tacrolimus | Neutral | 804.02 | 804.49 > 768.62 | 35 | 10 | 1.34 |
| Terfenadine | Base | 471.68 | 472.32 > 437.17 | 55 | 24 | 1.41 |
| Verapamil | Base | 454.61 | 455.35 > 190.16 | 35 | 24 | 1.33 |

a: Detected using electrospray in negative mode (method B)

**Supplemental material Table 4 Maximal rate of formation of testosterone hydroxylation and 4-nitrophenol glucuronide metabolites in intestinal microsome pools**

| **Pool** | **6β-OH TEST (pmol/min/mg)** | **16α-OH TEST (pmol/min/mg)** | **16β-OH TEST (pmol/min/mg)** | **Androstenedione (pmol/min/mg)** | **4-NP Glucuronide (nmol/min/mg)** |
| --- | --- | --- | --- | --- | --- |
| Pool 1 (Fresh)^a^ | ND | ND | ND | ND | ND |
| Pool 1 (FT)^b^ | 85.3 ±32.5 | 77.5 ±5.2 | 42.6 ±3.8 | 560.7 ±6.6 | 70.4 ±8.9 |
| Pool 2 (Fresh)^a^ | 113.3±30.9 | 55.1 ±13.1 | 61.2 ±4.3 | 429.9 ±30.9 | 84.8 ±28.6 |
| Pool 2 (FT)^b^ | 187.5 ±77.8 | 87.3±31.7 | 74.1 ±39.4 | 548.7±188.7 | 71.4 ±5.9 |
| Frozen (FT)^b^ | ND | ND | ND | ND | 13.0 ±5.3 |
| Scraping (FT)^b^ | 66.1±4.8 | 86.4 ±9.2 | 73.9 ±5.4 | 457.0 ±60.4 | 46.1 ±1.68 |
| Commercial HW^b^ | 190 ±53.5 | 86.9 ±4.1 | 121.7 ±11.8 | 314.5 ±55.3 | 56.1 ±4.1 |
| Commercial SD^b^ | 167.2 ±43.7 | 155.9 ±10.2 | 132.8 ±4.74 | 371.6 ±7.2 | 55.6 ±4.7 |

a: mean of 3 preparations in triplicate, b: 1 occasion in triplicate, Fresh: microsomes analysed on day of preparation before freezing FT: microsomes analysed following 1 FT cycle, ND: not determined
