# Supplementary figures and images for: Optimization of intestinal microsomal preparation in the rat: A systematic approach to assess the influence of various methodologies on metabolic activity and scaling factors
Source: Biopharm Drug Dispos. 2017 Apr 18;38(3):187–208. doi: 10.1002/bdd.2070 (PMC5413848; doi:10.1002/bdd.2070)

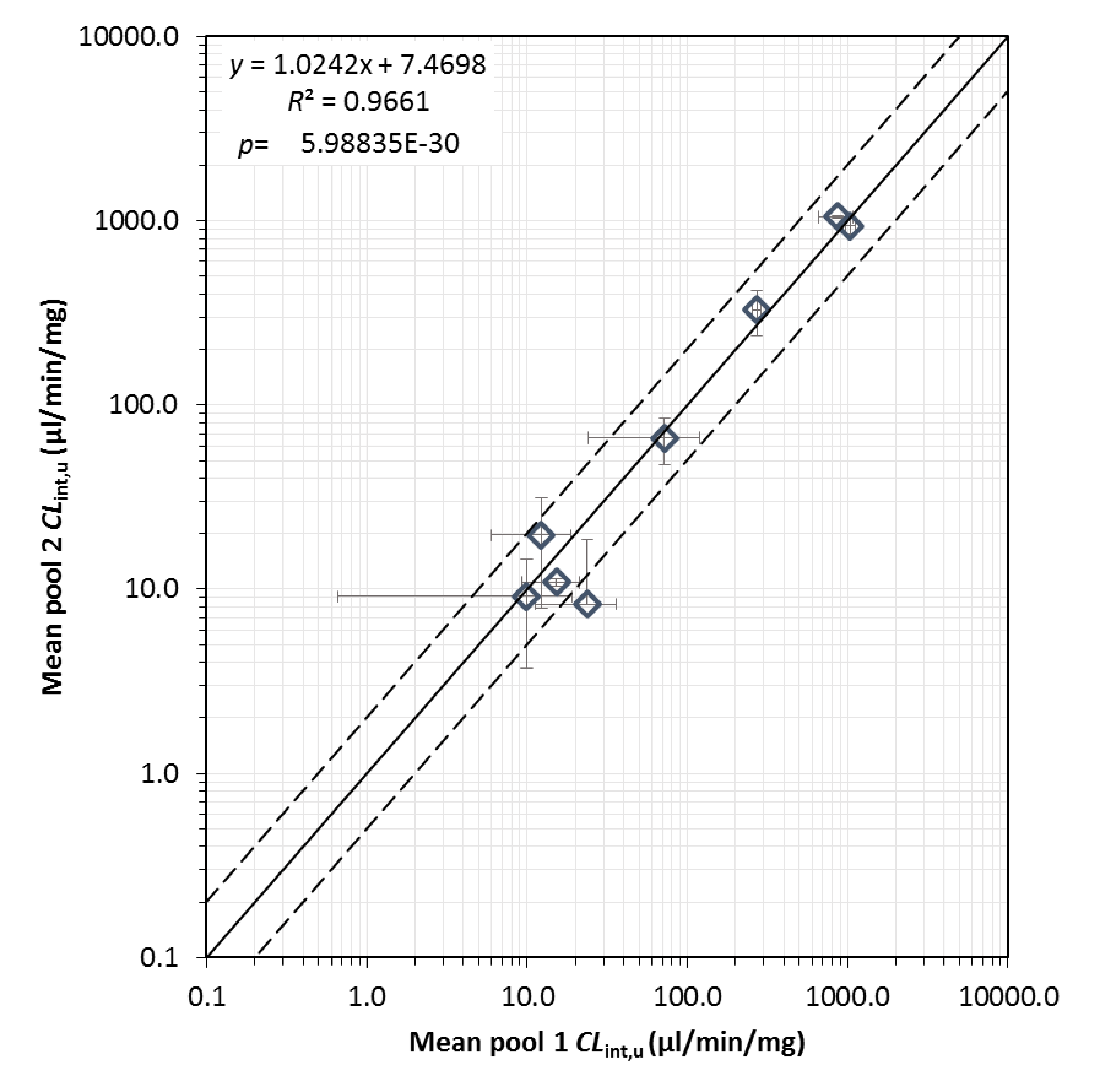

Supplement: Supplementary file 2 — Supplemental Table 1. Testosterone metabolites and respective rat cyp isoform. Supplemental Table 2. Testosterone hydroxy metabolite elution times and LLOQ. Supplemental Table 3. MS transitions for compounds in depletion studies in RIM, DIM, DLM and HIM, and pharmacokinetic studies in rat and dog blood and plasma. Supplemental Table 4. Maximal rate of formation of testosterone hydroxylation and 4‐nitrophenol glucuronide metabolites in intestinal microsome pools. [file BDD-38-187-s001.docx]
